# Single Center Experience of Eus-Guided Cystogastrostomy and Lumen-Apposing Metal Stent (LAMS) Positioning in Children with Pancreatic Fluid Collections: A Case Series

**DOI:** 10.3390/children11060643

**Published:** 2024-05-26

**Authors:** Annalisa Fiammetta Pasqualetto, Giovanni Boroni, Dario Moneghini, Filippo Parolini, Paolo Orizio, Anna Lavinia Bulotta, Guido Missale, Daniele Alberti

**Affiliations:** 1 Department of Pediatric Surgery, ASST Spedali Civili Children’s Hospital, 25123 Brescia, Italy; a.pasqualetto@unibs.it (A.F.P.); giovanni.boroni@unibs.it (G.B.); filippo.parolini@unibs.it (F.P.); paolo.orizio@asst-spedalicivili.it (P.O.); annalavinia.bulotta@asst-spedalicivili.it (A.L.B.); 2 European Reference Network for Hepatological Diseases (ERN RARE-LIVER); 3 Department of Digestive and Interventional Endoscopy, ASST Spedali Civili, 25123 Brescia, Italy; dariomoneghini@libero.it (D.M.); guido.missale@unibs.it (G.M.); 4 Department of Clinical and Experimental Sciences, University of Brescia, 25123 Brescia, Italy

**Keywords:** acute pancreatitis, pancreatic fluid collection, endoscopic cyst-gastrostomy, LAMS, walled-off necrosis

## Abstract

Pancreatic fluid collections (PFCs) are a well-known complication of pancreatitis. PFCs operative management includes percutaneous, endoscopic or surgical drainage. Even if in adult patients, endoscopic drainage is a well-established treatment, few data are available in pediatric setting. We report our single-center experience of EUS-guided cystogastrostomy and lumen-apposing metal stent (LAMS) positioning in children with PFCs; this, at the best of our knowledge, has never been reported before. All consecutive children with PFCs between April 2020 and November 2022 were enrolled in this retrospective study. PFCs were preoperatively evaluated with MRI or CT scan. All the procedures were performed under general anesthesia. A LAMS Hot-Axios^TM^ 10 × 15 mm was placed in all patients. We evaluated technical feasibility and clinical outcomes, including complications and recurrence rates. Follow-up included clinical observation, blood tests and US. EUS-guided cystogastrostomy was performed in 3 children (2 males; median age 13.2 years). Median maximum cyst diameter was 14.7 cm (range 10–22 cm). Technical and clinical success rates were 100%. No intra or post-operative complications occurred. Our experience suggests that this can be considered a safe and feasible treatment of PCFs even in the pediatric population, as long as the procedure is performed by an expert Endoscopist in a pediatric tertiary-level Center.

## 1. Introduction

Acute Pancreatitis (AP) is a relatively rare clinical condition in children, even if its incidence has increased in the last few decades (3.6–13/100,000 children-year) [1,2]. Unlike acute pancreatitis in adult population, natural history of pediatric acute pancreatitis is not clearly understood and there are limited data in literature, especially regarding its complications.

Pediatric acute pancreatitis has been classified by the NASPGHAN Pancreas Committee into mild AP, moderately severe AP, and severe AP according to pediatric-based criteria of organ dysfunction and systemic inflammatory response [2]. About 15% to 34% of pediatric patients develop severe disease, defined as “development of organ dysfunction that persists > 48 h. Persistent organ failure may be single or multiple and may develop beyond the first 48 h of presentation”.

In most children, acute pancreatitis resolves spontaneously with no consequences; nevertheless, in a small subset of patients, acute pancreatitis progresses, and local or systemic complications could occur [3,4].

Local complications are represented by pancreatic fluid collections (PFCs); according to 2012 revised Atlanta classification [5], PFCs can be described as acute peripancreatic fluid (APF), acute necrotic collections (ANC), pancreatic pseudocyst, and walled-off necrosis (WON). The distinction between them depends on the characteristics of the PCFs (fluid vs. presence of solid components) and the time range between AP and their formation (less or more than 4 weeks). Distinguishing these lesions is crucial because their natural history and management could be significantly different based on the type of PFC.

The prevalence of pseudocyst in children with acute pancreatitis ranges from 10% to 38% [6,7] and seems to be more common in pancreatitis following traumas, but their natural history and therapeutic indications have not been well reported in literature because data are scanty. Asymptomatic PFCs are usually managed conservatively; but in selective and/or symptomatic cases these patients should undergone drainage of PFCs [6,8].

Endoscopic approach is a well-established treatment of PFCs in the adult population [9,10], and it is mainly based on cystogastrostomy under endoscopic ultrasonography (EUS) guidance [9,10,11,12,13]. Nevertheless, these are technically challenging, time consuming, and demanding procedures; moreover, traditional tubular stents are associated with frequent risk of displacement, migration and stent occlusion, leading to recurrence of the collection [9,10].

Self expandable metal stents (SEMSs) and fully covered SEMS (FCSEMSs) have been proposed with the hope that a large luminal diameter would facilitate a more effective and lasting drainage. Unfortunately, FCSEMSs may migrate too and have been reported to cause tissue injury and bleeding [14]. To overcome these issues, new kind of stents have recently been released: lumen-apposing metal stents (LAMSs) are fully covered, braided stent with dumbbell configuration that are deployed under endoscopic ultrasound or fluoroscopic guidance with an electrocautery-enhanced delivery system [9,10].

In children, the role of endoscopic approach is still debated because of the limited data about safety and efficacy in this specific population [3,4,15,16,17] and caution should be exercised when extrapolating this approach to pediatric setting, as acute pancreatitis in children has different etiologies, clinical and radiological presentation compared with adults [18].

Aim of our study is to report our single-center experience with EUS-guided cystogastrostomy and LAMS positioning in children with PFCs. To the best of our knowledge, this device has never been reported in pediatric literature.

## 2. Materials and Methods

### 2.1. Population of the Study

All consecutive pediatric patients (<16 years old) with acute moderately-severe pancreatitis and pancreatic pseudocyst or walled-off necrosis managed in our Pediatric Surgery Department between April 2020 and November 2022 were retrospectively reviewed and enrolled in this series. Moderately-severe and severe acute pancreatitis were defined according to the NASPGHAN Pancreas Committee [2].

At admission, patients were pre-operatively assessed with abdominal ultrasound (US), abdominal MRI and/or CT scan. PFCs characteristics were noted and patients were included in the study if the MRI and/or the CT scan showed that the thickness of PFCs’ wall was greater than 0.1 cm and if the distance between the outer wall of the collection and the inner wall of the stomach was lesser than 1 cm. Patients with acute peri-pancreatic fluid and necrotic collections were excluded. Demographics, clinical and laboratory data, imaging, operative and follow-up results were collected from medical records and outpatient’s clinic visits.

### 2.2. Procedure

All the procedures were performed in the operative room with patients under general anesthesia and oro-tracheal intubation. Antibiotic prophylaxis with cephalosporin (cefazolin 25 mg/kg) was administered before the procedure. The procedure was performed by a senior endoscopist with high expertise in pediatric settings.

A Pentax^TM^ (Hoya Corporation, Tokyo, Japan) ultrasound video-gastroscope with 3.7-mm working channel was used. The LAMS used for cystogastrostomy was AXIOS^TM^ Stent and Electrocautery Enhanced (EC) Delivery System: HOT-Axios (Boston Scientific Corp, Marlborough, MA, USA), here referred to as EC-LAMS.

EC-LAMS is a self-expandible, nitinol braided FCSEMS with double flanges in a dumbbell shape. The stent is pre-loaded in an electrocautery-enhanced delivery catheter that is compatible with therapeutic echoendoscopes having a working channel of 3.7 mm diameter or larger. The nosecone at the catheter tip has a diathermic ring and cut wire. This device combines a cautery-enabled access catheter with the therapeutic stent. When fully expanded, the stent has a flange diameter twice that of the saddle section permitting apposition of the tissue layers, creating an “anastomosis” between two structures. According with the manufacturer’s instructions, the device should be wet with sterile saline and connected to electro-cautery, thus reducing the risk of bleeding.

**Figure 1:**
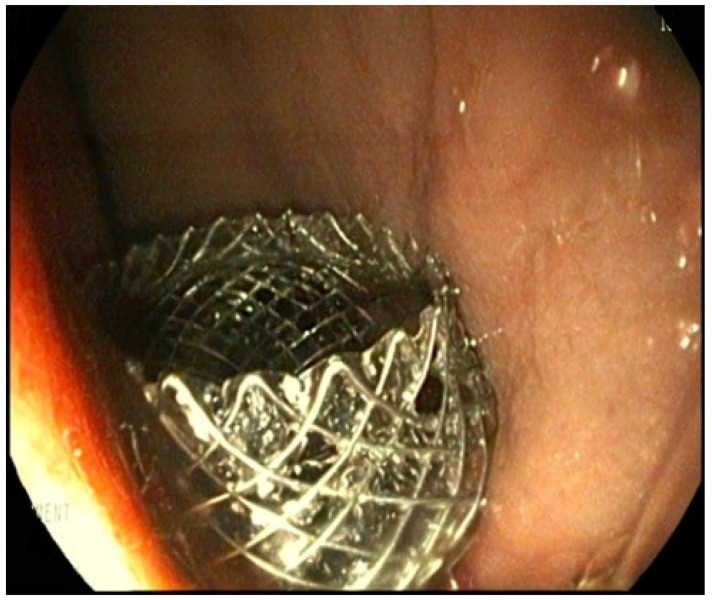
EC-LAMS 10 × 15 mm. Endoscopic image of the gastric flange of EC-LAMS at the end of its release into the gastric lumen.

The site of bulging of the PFC into the gastric lumen was endoscopically localized and then, the site of cystogastrostomy was identified by EUS. The procedure was carried out according to Boston Scientific indication: under EUS guidance, the catheter was advanced into the PFC and the stent deployed gradually; the distal flange in deployed into the PFC lumen and then retracted against the wall of the PFC; the proximal flange was deployed from the working channel into the gastric lumen under endoscopic guidance (Figure 1). No dilation of the stent was needed. At the end of the procedure, PFC content started to spontaneously drain into the gastric lumen, confirming the correct position of the stent.

**Figure 2:**
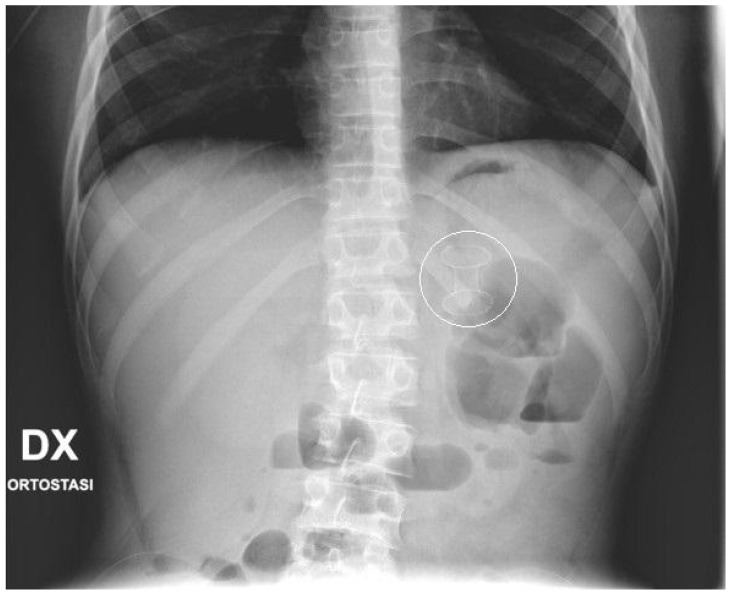
Final X-ray. X-ray performed at the end of the procedure that shows the EC-LAMS as a radiopaque dumbbell-shape object projected in the epigastrium.

No fluoroscopy was needed during the position of the stent; nevertheless, an X-ray was taken at the end of the procedure (Figure 2). In the following days, the correct position of the stent was confirmed with bed-side US. In the patient with WON, endoscopic necrosectomy sessions was not performed. Post-operative treatment included adequate pain control, supportive care and early enteral nutrition, according to the clinical condition of the patients. Complications occurring after the procedures, such as perforation, bleeding, stent displacement, were carefully documented. The stents were kept in place until resolution of the PFCs, assessed with pre-operative routine echography and/or MRI. When indicated, the stents were electively removed by a simple esophagogastroduodenoscopy, performed as an outpatient procedure.

### 2.3. Outcomes of the Study

Endoscopic technical success was defined as correct placement of EC-LAMS during the initial procedure. Clinical success was defined as clinical improvement and as radiological resolution of PFCs, confirmed by abdominal US and MRI. Intra-operative and post-operative complications were assessed according to the Clavien-Dindo Classification [19] and included migration and displacement of the stent and bleeding. PFC recurrence rates and need of re-interventions were also evaluated.

## 3. Results

In our department, we usually hospitalize a median of 3 children affected by moderately-severe pancreatitis per year. During the 31-months period of the study, three children (2 males) with a median age of 13.2 years (range 10–15 years) affected with moderately-severe acute pancreatitis, developed pancreatic pseudocyst or walled-off necrosis and were included in the series. Demographic and clinical data are resumed in Table 1. All patients were symptomatic; abdominal pain and persistent fever were present in all cases. Elevation of serum lipase activity was present in all 3 patients.

### 3.1. Patients in Details

#### 3.1.1. Patient n°1

**Figure 3:**
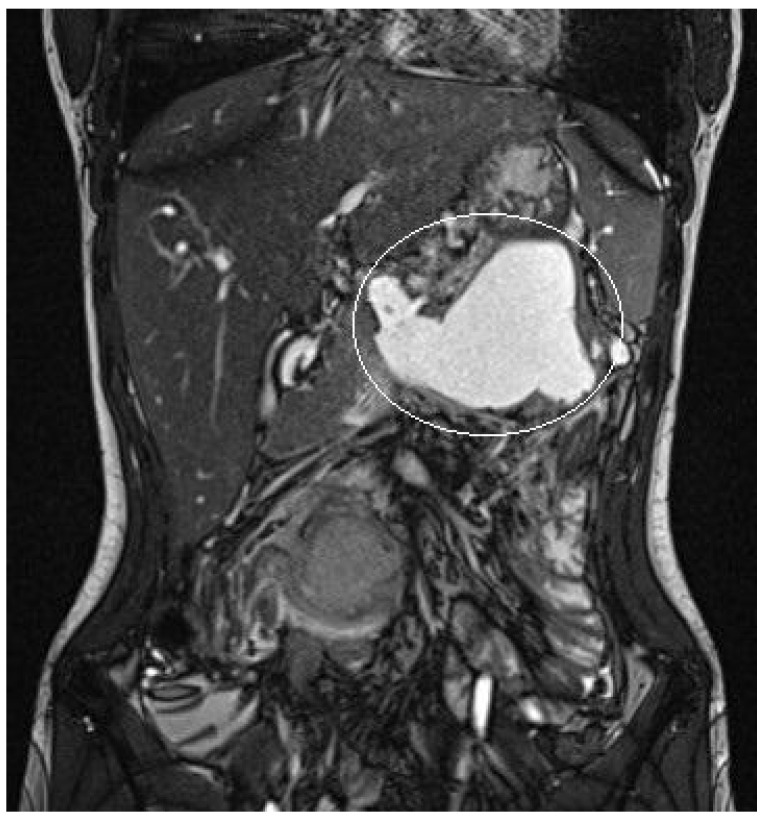
Patient n°1 MRI. Pseudocyst in the omental bursa with maximum diameter 10 cm.

Patient 1: A 15-year girl underwent elective surgery for a pancreatic solid pseudopapillary neoplasm: we performed a laparoscopic partial distal pancreatectomy with saving of the distal part of the pancreatic tail, that was not infiltrated by neoplasm. Few days after surgery, she developed a pancreatic leak with acute pancreatitis, with subsequent evidence of pancreatic pseudocyst. At preoperative MRI the pseudocyst had a major diameter of 10 cm (Figure 3). She initially underwent a first attempt of laparoscopic surgical debridement with placement of 2 tubular drains. As the patient still presented abdominal pain and vomit, US was performed, demonstrating an increase of the volume of the pseudocyst. After 8 days from the surgical procedure, the patient underwent EUS-guided cystogastrostomy and EC-LAMS placement as a rescue procedure. Post-operative course was uneventful and routine US performed after cystogastrostomy, showed a progressive resolution of the collection; retrogastric collection was no more detected 19 days after the procedure, suggesting its complete resolution. Twenty-one days after EC-LAMS positioning, we performed an endoscopy to remove the device; no complications occurred during or after the procedure.

After a total of 3 years and 8 months of follow-up, no recurrences occurred and the patient is well.

#### 3.1.2. Patient n°2

**Figure 4:**
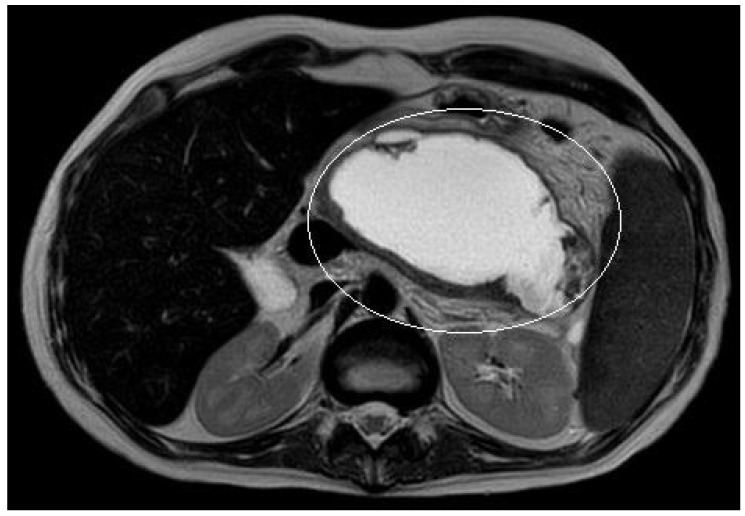
Patient n°2 MRI. Pseudocyst with maximum diameter 12 cm in the left hypochondrium in close proximity to the posterolateral wall of the stomach.

A 15-year boy was affected by B-acute lymphoblastic leukemia and severe coagulopathy; he developed a chemotherapy-induced acute cholecystitis, acute pancreatitis complicated by septic shock and PFC. At MRI the major diameter of the collection was 12 cm (Figure 4). After unsuccessful attempts of conservative treatment, the boy underwent two different attempts of EUS-guided trans-gastric pseudocyst puncture and liquid aspiration, within a month between the two procedures. few days after the second one, the PFCs persisted and the boy still presented abdominal pain and fever. As the need to not delay chemotherapy was pressing, the patient underwent EUS-guided cystogastrostomy and EC-LAMS positioning as a rescue procedure. Post-operative course was uneventful. MRI performed 14 days after the procedure showed a significant reduction of the pseudocyst, which was no longer visible on the ultrasound performed right before its removal. EC-LAMS stayed in place for a total of 28 days and was endoscopically removed, without any intra-operative or post-operative complication.

During 1 year and 3 month of follow-up, no recurrence of acute pancreatitis and pseudocyst occurred; the patient died after lymphoma relapse.

#### 3.1.3. Patient n°3

**Figure 5:**
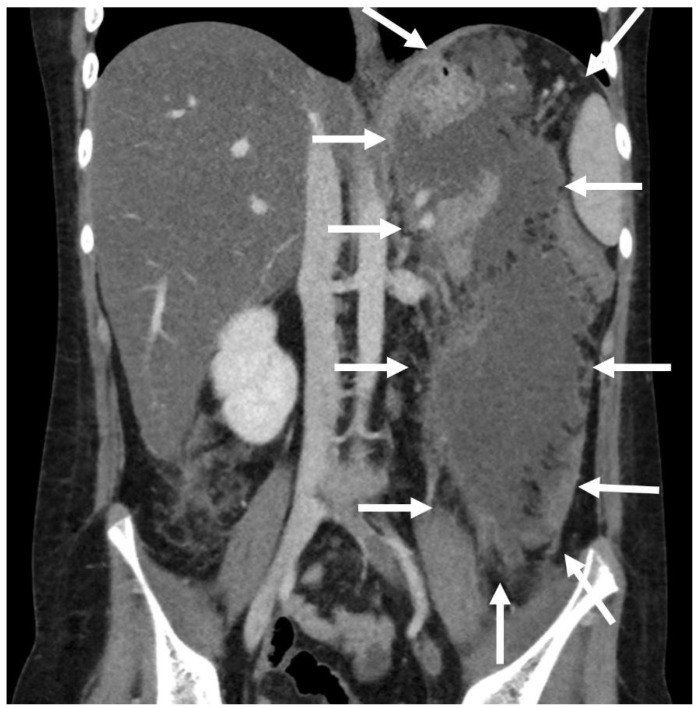
Patient n°3 CT. Necrotic-hemorrhagic pancreatitis (body/tail origin) with voluminous walled-off necrosis developing in the left quadrant (hypochondrium/flank; relations to stomach, spleen, kidney, descending colon), extending craniocaudal about 22 cm.

**Figure 6:**
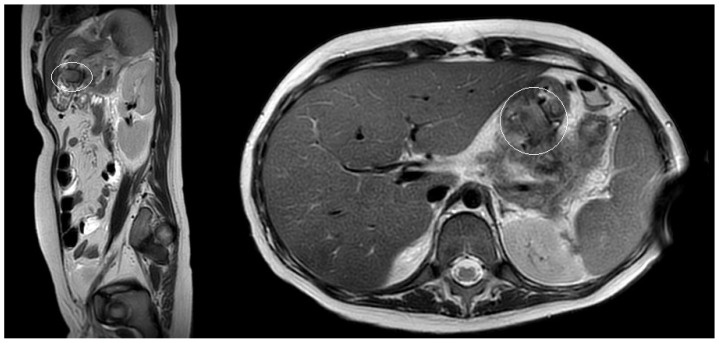
Patient n°3 MRI. Reduction of the walled-off necrosis; the device is in place (in the white circle).

A 10-year boy affected by T-lymphoblastic lymphoma developed a WON after chemotherapy-induced necrotic-hemorrhagic pancreatitis, with fever, vomit and abdominal pain. Clinical conditions were also complicated by deep vein thrombosis involving the left femoral vein and inferior vena cava. Abdominal CT showed a large WON, with a major diameter more than 22 cm (Figure 5). Considering the worsening of clinical condition and the need to an early resume of the chemotherapy, he underwent a primary EUS-guided cystogastrostomy and EC-LAMS positioning; we did not perform necrosectomy. Post-operative course was uneventful. MRI performed 37 days after the procedure, showed a significant reduction of the WON (Figure 6); subsequent US images were performed for follow-up, until the WON was no more visible. Endoscopic removal of the device was performed 48 days after cystogastrostomy.

Patient is still undergoing oncologic therapy, but at one-year follow-up he has had no recurrence of acute pancreatitis and PFCs.

### 3.2. Results

Results are summarized in Table 2.

The median diameter of PFCs was 14.7 cm, ranging from 10 to 22 cm. In all cases the 10 × 15 mm-length EC-LAMS was positioned. This device has an internal lumen of 15 mm-length, a saddle length of 10 mm and the diameter of the flange is 24 mm; this is compatible with therapeutic EUS scopes with working channel 3.7 mm or more.

Median procedure time from patient positioning on the operative table, to final abdominal X-ray (Figure 2) was 37 min (range 33–40 min). EC-LAMS were removed endoscopically as an elective outpatient procedure, after a median time of 32 days (range 21 to 48 days), after confirming resolution of the PFCs at imaging. No patient required multiple endoscopic aspirations through the device.

Technical and clinical success were reached in all cases; no intra-operative complications occurred. No early or late complications were observed. At a 3-years and 8 months maximum follow-up, acute pancreatitis and PFCs did not recur in any patient.

## 4. Discussion

Despite the increasing incidence of acute pancreatitis in the pediatric population in the last few decades, there are no specific pediatric therapeutic guidelines for this severe disease, but only recommendations and reviews that often rely on adult experience [3,4,18]. Incidence of AP is estimated 3.6–13/100,000 children-year [1,2] and around 10 to 38% of children affected develop pseudocyst [6,7].

In the adult population, acute pancreatitis is a well-known condition with a higher incidence and a better understood physiopathology also regarding its complications. Twenty percent of adult patients diagnosed with acute pancreatitis, have moderately severe or severe AP with local complications [20]. In literature, the incidence of acute PFC is estimated between 38.6–48.3% [21,22,23] and the incidence of pseudocyst around 6% [21,22].

The majority of adult patients with acute PFC do not need any kind of intervention because collections will resolve spontaneously; drainage is indicated in case of symptoms [24] such as worsening abdominal pain, persistent nausea or vomiting, weight loss. Size alone does not represent an indication for treatment, however pseudocyst larger than 6 cm are more likely to be symptomatic [25].

Historically, the management of PCF has primarily been surgical. With the development of new endoscopic techniques and improvement in the endoscopic approach, in the last few decades this has become the approach of preference in adult field [13,24,26,27]. A randomized controlled trial of Bang and coll. Ref. [26] shows that endoscopic approach should be preferred over minimally invasive surgery in treatment of adult patients with infected necrotizing pancreatitis.

The traditional endoscopic approach is based on endoscopic puncture of the PFC seen as a prominent bulge into the gastric lumen and creating a fistula through the gastric wall via insertion of one or more stents.

Introduction of EUS-guided approach has made it possible to extend the indication to PFC that has not an evident bulge into the gastric lumen, which represents almost half of all cases [27]. Furthermore, EUS allows to clearly identify PCF, measure it, and evaluate the distance between it and the gastric wall. It reduces risk of complications such as puncture of other structures as blood-vessels thanks to the possibility of identifying them [28,29].

Another important evolution of the technique concerns the type of stents used: plastic stents or Self Expandable Metal Stent (SEMS) and Fully-Covered SEMS (FCSEMS) [12,13,28,29,30]. Plastic stents have a smaller diameter and their configuration predispose them to the risk of occlusion and migration. Cystogastrostomy with plastic stents generally requires performing a dilation of the channel and is often necessary to insert multiple plastic stents to drain PFC effectively; the procedure can become time-consuming and can increase risk of complications. They do not enable to perform debridement of necrotic collections or necrosectomy.

SEMS and FCSEMS were specifically designed to overcome these limits: they have a larger lumen improving drainage and decreasing risk of stent occlusion. They may also decrease the risk of leakage between PFC and gastric lumen, and they may reduce risk of bleeding thanks to the compression they make.

The recent alternative device is represented by LAMS, as the EC-LAMS used in our experience. The unique configuration of LAMS reduces risk of migration, dislocation and leaks approximating the two walls (gastric and PFC’s ones). The larger lumen of the device decreases risk of occlusion and allows to perform therapeutic procedures through it, like fluid aspiration and necrosectomy.

The delivery system enables single operator, single-hand control for the entire procedure and reduces the number of device exchanges. The cautery system reduces risk of intra-operative bleeding and eliminates need of preliminary dilatation. For all this reasons, LAMS are more likely to reduce procedure time.

A recent meta-analysis of Yoon et coll. et al. [31] showed that metal stents are superior to plastic stents for PFC drainage both in terms of clinical success and adverse events.

The difference between these devices is important especially when they are used to treat WON: in a retrospective cohort study that compares the 3 devices (plastic stent, FCSEMS and LAMS), the rate of complete resolution of WON was lower with plastic stents compared with the other two; LAMS showed to be more efficient in reducing need of reinterventions and further procedures [32].

So, in adults, EUS-guided cystogastrostomy can now be considered the first-line treatment of choice for PFCs [11,28,31]. However, in the pediatric population, concerns arise regarding technical issues related to the size of therapeutic EUS-scopes, besides the fact that solid data regarding the safety and efficacy of EUS-guided drainage in children are scanty. Furthermore, these data are completely missing concerning the employment of FCSEMS and LAMS.

In the majority of pediatric patients, PFCs do not need any kind of drainage and resolve spontaneously; that happens more commonly in children than adults, especially if <5 cm in size. Therapeutic interventions are indicated in symptomatic cases and in cases of infected collections [18].

When indicated, PFC drainage in children is often based on adult patient experience because literature in pediatric field is scanty. While in recent years, endoscopic drainage has been demonstrated feasible in children, EUS is still hardly used perhaps due to lack of experience and data [8,15]. To the best of our knowledge, we here describe the first case series in literature of EUS cystogastrostomy and EC-LAMS positioning in children with PFC caused by moderately-severe acute pancreatitis.

In our experience, need of PFCs drainage was limited to selected cases: symptomatic patients, failure of previous treatments with persistence of PFC, higher risk of complication such as infection, thrombosis, or bleeding from venous and/or arterial vessels next to the collection. Other important inclusion criteria for EC-LAMS placement were: the thickness of PFCs’ wall greater than 0.1 cm and the distance between the outer wall of the collection and the inner wall of the stomach lesser than 1 cm.

In some cases, patient’s comorbidity could represent an indication of urgency, as in our series in which patients 2 and 3, respectively affected with B-acute lymphoblastic leukemia and T lymphoblastic lymphoma, needed an early resumption of chemotherapy.

In our experience, we decided to perform cystogastrostomy using EC-LAMS as a rescue procedure in patients 1 and 2, in which previous therapeutic options had failed.

Cystogastrostomy and EC-LAMS positioning was however a primary treatment for the third patient: we made this choice after multidisciplinary evaluation of the critical oncological and medical condition of the child that required rapid resolution of WON, and as the result of our positive experience with this device in previous PFCs.

It is important to state that cystogastrostomy and EC-LAMS positioning remains a difficult procedure to manage, that requires a lot of technical expertise to reduce risk of complications and recurrence; therefore, it has to be performed in a tertiary-level Center by an expert endoscopist with experience in the pediatric field.

Limitation of our study is represented by the small number of patients included and the heterogeneity of the group, and the retrospective nature of the study which has inherent limitations.

## 5. Conclusions

Acute Pancreatitis and its complications, while still remaining a rare disease in the pediatric population, has been subject to increase in incidence in the last few decades. Currently there are no specific pediatric therapeutic guidelines for the management of PFC, but only recommendations and reviews that often rely on adult experience.

To the best of our knowledge, this represents the first series reported in literature of cystogastrostomy and EC-LAMS positioning in pediatric patients affected by PCF as a result of an acute moderately-severe pancreatitis.

Our experience, although limited, suggests that EC-LAMS positioning can be considered a safe and feasible procedure in selected children with symptomatic PCF. Compared to traditional stents, EC-LAMS may offer a higher rate of success with reduced number of procedures and a faster recovery. In patients with comorbidities, such as in our series, this could be a significant advantage. In our experience, the procedure could be offered as first line treatment (such as in our patient n°3), or as a rescue procedure (such as in patients n°1 and 2°).

This procedure, nevertheless, is still technically challenging and we recommend being performed only by an expert Endoscopist in a tertiary-level Center, in order to reduce adverse events and recurrence rate.

Pediatric randomized controlled trials would be necessary to compare efficacy and safety of this procedure with alternative drainage methods, but they are difficult to obtain due to the limited number of pediatric cases.

## Figures and Tables

**Table 1:** Patient’s characteristics and outcomes.

	Pt 1	Pt 2	Pt 3
**Sex**	F	M	M
**Age**	15	15	10
**PFC**	Pseudocyst	Pseudocyst	WON
**PFCs etiology**	After pancreatic surgery—pancreatic solid pseudopapillary neoplasm	Chemotherapy-induced pancreatitis—B-acute lymphoblastic leukemia	Chemotherapy-induced pancreatitis—T-lymphoblastic lymphoma
**PFCs max size**	10 cm	12 cm	22 cm
**Procedure**	Rescue	Rescue	Primary

**Table 2:** Caption.Results.

	Pt 1	Pt 2	Pt 3
**PFCs max size**	10 cm	12 cm	22 cm
**EC-LAMS**	10 × 15 mm	10 × 15 mm	10 × 15 mm
**Median procedure time**	33 min	38 min	40 min
**EC-LAMS removing time**	21 days	27 days	48 days
**Need of further procedures**	None	None	None
**Intra/post-op complications**	None	None	None
**Follow-up**	No recurrence	No recurrence	No recurrence

## Data Availability

The data presented in this study are available on request from the corresponding author. The data are not publicly available due to privacy restriction.
